# Factors associated with depression among HIV/AIDS children in China

**DOI:** 10.1186/s13033-019-0263-1

**Published:** 2019-02-20

**Authors:** Enpeng Zhou, Zhengxue Qiao, Yuewu Cheng, Jiawei Zhou, Wenbo Wang, Mingzhe Zhao, Xiaohui Qiu, Lin Wang, Xuejia Song, Erying Zhao, Ruopeng Wang, Xueyan Zhao, Yanjie Yang, Xiuxian Yang

**Affiliations:** 1 Psychology Department of the Public Health Institute of Harbin Medical University, No. 157, Baojian Road, Nangang District, Harbin, 150081 Heilongjiang Province China; 2 Shangcai Center for Disease Control and Prevention, Zhumadian, Henan China

**Keywords:** HIV-infected children, Depression, Psychosocial

## Abstract

**Background:**

Depression in HIV/AIDS children not only worsens the progression and outcome of illness, but also impacts their quality of life, having a negative influence on society. The present study was conducted from a psychosocial perspective, considering children’s social desirability, cognitive emotion regulation, and perceived social support to identify the factors influencing depression in HIV-infected children in China.

**Methods:**

Participants were 155 children aged 8–18 years who were eligible to participate in this study assessing depression and associated risk factors using the Children’s Depression Inventory, Cognitive Emotion Regulation Questionnaire, Multidimensional Scale of Perceived Social Support, and Children’s Social Desirability scale. Hierarchical linear regression analysis was conducted to model the effects of social desirability, perceived social support, and cognitive emotion regulation on depression in HIV/AIDS children.

**Results:**

Statistically significant linear relationships were found among social desirability, perceived social support, partial dimensions of cognitive emotion regulation, and children’s depression scores. Perceived social support, planning and positive reappraisal were negatively related to the depression. Conversely, social desirability, catastrophizing and other-blame were positively associated with the depression. Linear regression analysis indicated that children’s social desirability, perceived social support, and one dimension of cognitive emotion regulation (catastrophizing) were found to significantly predict depression.

**Conclusions:**

Psychosocial factors have an important influence on the depression experienced by HIV-infected children. Interventions from personal subjective psychosocial to reduce depression in HIV-infected children in China are warranted.

## Introduction

Human immunodeficiency virus (HIV) infection has become a significant social emergency worldwide. HIV is threatening children as never before. According to the UNAIDS, 1.8 million children (< 15 years) were HIV positive at the end of 2017 [1]. Although the epidemic trend of global HIV infection was declining, children represent a growing share of people living with HIV worldwide and China is no exception [2]. Children infected by HIV not only suffer from the heavy burden of physical disease associated with chronic illness but are also at risk of developing mental health disorders. Depression is the most common neuropsychiatric complication in HIV-infected children and may occur during all phases of the infection [3]. Depression in HIV-infected children leads to a negative impact on a wide range of aspects of their life quality and has a profoundly deleterious influence on society [4–6]. Previous studies have shown that HIV-infected children suffering from depression are more likely to develop maladaptive outcomes and behaviors, including dropping out of school, drug abuse, alcohol use, and engaging in high-risk sexual behaviors that increases the risk of HIV transmission [7, 8]. Depression also has a wide range of negative effects on the progression of illness in HIV-infected children. It decreases their immune status and adherence to antiretroviral therapy, which may result in the potential development of drug resistance and decreased clinical effectiveness [9, 10], increasing the disease cost burden and mortality associated with illness [11–13]. Therefore, it is imperative to explore the factors influencing the development of depression in HIV-infected children.

Childhood is full of challenges that children have to deal with, including pressures coming from academic study, the relationships with peers, changes in the body and from the development of cognition function. Children who are early in psychological developmental stages, including cognition, are more sensitive to stressful events and more susceptible to the impact of social desirability [14, 15]. HIV-infected children may suffer from more stressors caused by a higher exposure to traumatic life events which result in depression, in addition to those events experienced by healthy children. Many of these stressors are chronic and impact on multiple levels of children’s lives. Despite the prevalence of HIV being low in China, levels of stigma remain widespread and profound [16, 17]. HIV-infected children are confronted with stereotypes, discrediting, prejudice, and discrimination caused by HIV infection-related stigma that may lead to them being isolated, helpless, and bullied [18, 19]. HIV-infected children face negative impacts in many ways within society. In terms of coping with stigma and integrating into society, HIV-infected children may also be affected by social desirability. Social desirability can be viewed as a tendency to present oneself favorably or to obtain approval by responding in a culturally and socially acceptable manner [20]. Studies have shown that social desirability correlates with depression in normal children but findings are heterogeneous [21, 22]. No known studies to date have explored the effects of social desirability on responses to depression in HIV-infected children. In addition to the pressure of stigma, HIV-infected children may suffer as a result of illness experienced by their parents, poor relationships with their parents, and even the loss of their parents. More importantly, the treatment and progression of HIV infection impact upon on HIV-infected children. The persistent use medication over a long time and uncertain trajectory of their illness likely make children susceptible to depression. When facing one or more of these above stressors in conjunction with the heavy burden of stressors associated with HIV-infection, social support is an important factor in ameliorating depression among HIV/AIDS patients. Perception of social support is a subjective aspect of social support, referring to the emotional experience and satisfaction of being respected, supported, and understood. Indeed, the perception of social support can have a directly beneficial impact on mental health [23, 24] and buffer against suicide risk created by stressful life events [25]. Cognitive emotion regulation is also an important factor. Studies have illustrated that emotion regulation plays a significant role in the mental health of children [26, 27]. Children learn to regulate their emotions by means of cognitions or thoughts about themselves and/or others. Cognitive emotion regulation refers to the cognitive means of handling the intake of emotionally arousing stimulations [28]. Studies have shown that using different cognitive emotion regulation strategies has different outcomes [29, 30]. The strongest relationships have been found between the cognitive emotion regulation strategies of rumination, catastrophizing, and self-blame and the reporting of symptoms of depression [31–34]. These findings might imply that by using these strategies, children may be more vulnerable to developing symptoms of depression in response to negative life events than others [34].

Depression impacts on the progression of illness and quality of life in HIV-infected children resulting in outcomes that increase the social burden of disease, as well as the diffusion and mortality of HIV/AIDS. Despite the increased interest in this field and the publication of studies highlighting the high levels of depression in children, there continues to be a dearth of data concentrating on the personal subjective psychosocial perspective. Research is needed to examine the associated influencing factors of depression in HIV-infected children, especially in the Chinese socio-cultural context. Previous studies exploring depression in HIV-infected children are mostly from the perspectives of society and family. We explored the roles of children’s social desirability, cognitive emotion regulation, and perceived social support to identify the factors influencing of depression in HIV-infected children in China. It was hoped that this would provide a basis for developing measures to reduce the levels of depression and effectively improve life conditions for HIV-infected children.

## Methods

### Study sites and participants

The current study was conducted in a county of the Henan Province, named Shangcai, an area with one of the highest incidences of HIV-infection in China. Here, a large number of rural residents were affected with HIV through unhygienic blood collection practices in the late 1980s and early 1990s. By the end of 2014, there were 64,663 people living with HIV in Henan Province (500,679 in China), for whom 34,150 (204,683 in China) the disease had progressed to AIDS. Although official prevalence data were not available for Shangcai county, local epidemiological surveys report that HIV infection rates were as high as 9.1–15.3% in some of the villages in this county. We obtained village-level HIV surveillance data from the anti-epidemic station to screen the names of HIV-infected children. Sample selection took place using randomized stratified cluster sampling from 43 of 71 schools in the district. The participants aged 8–18 years old were eligible to participate in and all of them were currently in school (from primary to senior high level). After excluding ten invalid questionnaires (those with > 20% questions unanswered), 145 participants returned suitable questionnaires.

### Survey procedure

We contacted the headmasters of local schools with the help of the Center for Disease Control and the Prevention officer. The headmasters in each school called the HIV-infected children together. To protect participants’ privacy, the headmasters worked with children to come up with an individualized plan for assessment in terms of the preferable time and place for them to feel comfortable to meet with the researchers. Once the time and place were confirmed, interviewers accompanied by the headmasters visited the child and provided them with a detailed description of the study design and potential benefits and risks (including confidentiality issues). Informed written consent was sought from the children (> 12 years old) or their guardians (8–12 years old). In situations where no guardians were available to provide consent, a “resource person” was identified for each of these children as a means of protection. The resource persons included community leaders, caregivers, or school headmasters. The children and their guardians were assured of confidentiality. For participants with limited literacy, it was necessary to provide clarification or instruction when needed. The interviewers were trained education and psychology graduate students. It took about 1 h to complete the entire assessment inventory, including taking breaks. The questionnaires were collected immediately when completed. We checked the questionnaires to avoid errors and ensure data quality and provided a gift as a token of appreciation for participation. This study was approved by the Ethics Committee of Harbin Medical University.

### Measures

#### Demographic characteristics

Children were asked to report on individual and family characteristics. These characteristics including the participants’ name, gender, age, ethnicity, drugs of abuse, major field of study, grade level, the number of family, satisfaction with their major, parental relationship, mother’s and father’s education levels, mother’s and father’s employment status, parental living conditions and their family’s economic situation.

#### Children’s Depression Inventory

Depression was measured using the 27-item Children’s Depression Inventory (CDI) [35]. A three-factor model was proposed for the Chinese version of the CDI [36]. Items are scored from 0 to 2, with a higher score indicating a greater level of symptom severity. The sum of the score ranges from 0 to 54, and a cut-off score of 19 was used to identify children in the upper 10% in samples of nonclinical children [37]. The Cronbach’s alpha of the scale was 0.82 in the present study.

#### Cognitive emotion regulation strategies

Cognitive emotion regulation strategies were measured by the Cognitive Emotion Regulation Questionnaire (CERQ) [38]. The CERQ includes nine conceptually distinct scales. These scales all consist of two items referring to what people think after the experience of threatening or stressful life events, ranging from 1 (almost) never) to 5 ((almost) always). A subscale score can be obtained by adding up the two items, the minimum score is 0 and the maximum score is 8. The higher the subscale score, the more the specific cognitive strategy is used. The following cognitive emotion regulation strategies were measured: self-blame, referring to thoughts of putting the blame of what you have experienced on yourself; other-blame, referring to thoughts of putting the blame of what you have experienced on others; rumination or focus on thought, referring to thinking about the feelings and thoughts associated with the negative event; catastrophizing, referring to thoughts of explicitly emphasizing the terror of an experience; putting into perspective, referring to thoughts of playing down the seriousness of the event or emphasizing the relativity when comparing it to other events; positive refocusing, referring to thinking about joyful and pleasant issues instead of thinking about the actual event; positive reappraisal, referring to thoughts of attaching a positive meaning to the event in terms of personal growth; acceptance, referring to thoughts of accepting what you have experienced and resigning yourself to what has happened; and planning, referring to thinking about what steps to take and how to handle the negative event. The CERQ has been shown to have good reliability and validity in China [39]. Cronbach’s alpha for the whole scale was 0.86 in the present study.

#### Children’s Perceived Social Support

The perceived social support measure was adapted from the Multidimensional Scale of Perceived Social Support (MPSS) scale [40], which has been validated in children and adolescents [41, 42]. The original MPSS scale included three subscales assessing the source of emotional support (family, friends, or significant others). Considering the potential role of school teachers and students in supporting children affected by AIDS in rural China, we created a parallel subscale for teacher and student support using similar questions instead of support using a 7-point response option (ranging from 1 =  “very strongly disagree” to 7 =  “very strongly agree”). Cronbach’s alpha of the whole scale was 0.88 in the present study.

#### Children’s social desirability

The Marlowe–Crowne Social Desirability scale was used as a model by Crandall et al. to develop the Children’s Social Desirability scale [43]. The version has 48 true–false items. Answers that matched the socially desirable choice were scored as 1 point. Possible scores ranged from 0 to 48 with higher scores indicating a higher tendency toward socially desirable responding. The Cronbach’s alpha of the whole scale was 0.88 in the present study.

### Data analysis

The Statistical Package for Social Sciences 18.0 (SPSS 18.0) program was used for statistical analysis. All tests were two-tailed and the significance level was set at p < 0.05. Differences between the groups were tested by Chi square test. In addition, Pearson correlation analyses were conducted to examine the strength of associations between all variables. Linear regression analysis was conducted to model the effects of social desirability, perceived social support and cognitive emotion regulation on depression in HIV/AIDS children. The nine subscales of measure of cognitive emotion regulation were regarded as nine variables. Thus, we assessed all variables thought to be potentially correlated with depression based on previous studies. The initial bivariate correlations analysis identified six variables thought to be possibly associated with depression. The six candidates with p values < 0.05 were entered into the multivariate regressions. And we used step-wise fashion in the linear regression.

## Results

### Characteristics of the study sample

A total of 47 participants (32.41%) scored above the threshold for depressive symptoms. Of the 145 participants, 83 (57.2%) were males and 62 (42.8%) were females. The average age of the participants was 16.01 years (standard deviation (SD) = 1.929), with a range of 11–18 years. The numbers of participants in each grade were as follows: primary school, 17; senior school, 87; junior school, 28; and others, 13. Of the 145 children, 70 had lost one or both parents and 75 still had both parents alive. No significant differences in the CDI scores were found between gender, age, grades, or condition of parents (Table 1).

**Table 1:** Participant demographic data and CDI scores

Group	N (%)	Depression	Non-depression	X^2^	p
Gender					
Male	83 (57.2)	28	55	0.155	0.694
Female	62 (42.8)	19	43		
Age					
11–14	30 (20.7)	7	23	1.424	0.233
15–18	115 (79.3)	40	75		
Grade					
Primary	17 (11.7)	6	11	2.761	0.430
Junior	87 (60)	29	58		
Senior	28 (19.3)	6	22		
Other	13 (9)	6	7		
Conditions of parents					
Loss	75 (51.7)	23	52	0.216	0.642
Alive	70 (48.3)	24	46		

### Bivariate correlations among variables

Statistically significant linear relationships were found among social desirability, perceived social support, planning, positive reappraisal, catastrophizing, other-blame factors, and Chinese HIV/AIDS children’s depression scores (p < 0.05). Among these variables, perceived social support (r = − 0.456, p < 0.01), planning (r = − 0.209, p < 0.05) and positive reappraisal (r = − 0.243, p < 0.01) were negatively related to the level of depression. Conversely, social desirability (r = 0.423, p < 0.01), catastrophizing (r = 0.277, p < 0.01) and other-blame (r = 0.203, p < 0.05) were positively associated with the level of depression. There were no significant correlations among self-blame, acceptance, rumination or focus on thought, rumination or focus on thought, positive refocusing, putting into perspective and depression (Table 2).

**Table 2:** Correlation coefficients of variables

Variables	1	2	3	4	5	6	7	8	9	10	11	12
1. Depression	1											
2. Social desirability	0.423 **	1										
3. Perceived social support	− 0.456**	− 0.313**	1									
4. Self-blame	− 0.118	− 0.121	0.237**	1								
5. Acceptance	− 0.040	0.204*	0.050	0.612**	1							
6. Rumination or focus on thought	− 0.151	0.048	0.110	0.532**	0.514**	1						
7. Positive refocusing	0.083	0.253**	− 0.073	0.325**	0.405**	0.453**	1					
8. Planning	− 0.209*	− 0.121	− 0.356**	0.490**	0.462**	0.489**	0.282**	1				
9. Positive reappraisal	− 0.243**	− 0.236**	0.351**	0.500**	0.452**	0.444**	0.313**	0.767**	1			
10. Putting into perspective	− 0.118	0.087	0.220**	0.457**	0.459**	0.552**	0.432**	0.550**	0.501**	1		
11. Catastrophizing	0.277**	0.244**	− 0.162	0.318**	0.236**	0.314**	0.447**	0.255**	0.194*	0.426**	1	
12. Other-blame	0.203*	0.195*	− 0.08	0.417**	0.351**	0.291**	0.368**	0.243**	0.185*	0.346**	0.512**	1

*p < 0.05, **p < 0.01

### Multivariate analysis of associations between depression and other variable

In the linear regression, the three variables (planning, positive reappraisal and other-blame) were excluded from the model. In the first model the perceived social support accounted for 20.8% of the variance in the depression total scores (F change = 37.56), and perceived social support (β = − 0.456, t = − 6.129, p < 0.001) was significantly to predict depression. In the second model, the explaining variance increased to 29.5% (F change = 17.592), perceived social support (β = − 0.359, t = − 4.836, p < 0.001), and social desirability (β = 0.311, t = 4.194, p < 0.001) were significantly to predict depression. In the final model, the explaining variance increased to 31.7% (F change = 4.531), and perceived social support (β = − 0.344, t = − 4.678, p < 0.001), social desirability (β = 0.278, t = 3.714, p < 0.001) and catastrophizing (β = 0.153, t = 2.129, p = 0.035) were significantly to predict depression (Table 3).

**Table 3:** Linear regression analysis of the relationships between social desirability, perceived social support, catastrophizing, and depression total scores

	β	t	p	F change	R^2^	R^2^ change
Model 1				37.560	0.208	0.208
Perceived social support	− 0.456	− 6.129	< 0.001			
Model 2				17.592	0.295	0.087
Perceived social support	− 0.359	− 4.836	< 0.001			
Social desirability	0.311	4.194	< 0.001			
Model 3				4.531	0.317	0.022
Perceived social support	− 0.344	− 4.678	< 0.001			
Social desirability	0.278	3.714	< 0.001			
Catastrophizing	0.153	2.129	0.035			

## Discussion

The findings of this study support psychosocial factors have an important influence on the depression in HIV-infected children. Among the three influential factors, the strongest indicator of depression was perceived social support. We found that the higher levels of perceived social support are associated with lower levels of depression, consistent with the global literature on the “buffer” function of perceived social support. Studies have found that lower perceived adequacy of social support has been linked to poor mental health [44, 45]. Similarly, Bal et al. [46] revealed that a higher perceived availability of social support was found to be directly associated with fewer symptoms related to trauma among a group of adolescents who had suffered a stressful event. Importantly, in a study among individuals living with HIV/AIDS, Blaney et al. [47] reported that the perceived availability of social support was a strong predictor of reduced depression. This in line with other studies showing that people with HIV who reported greater satisfaction with their social support had lower rates of depression [48] and greater perceived social support was associated with better mental health [49]. The possible explanation for this is that the perception that one is accepted and valued in one’s interpersonal environment bolsters esteem, confidence, and efficacy, which guards against depression. Social resources are generally protective against adverse psychological responses to stressful situations [50]. The perceived availability of social support may prevent or reduce the occurrence of depressive symptoms by preventing a potential stressor from being perceived as stressful. HIV-infected children may experience a greater number of stressful situations caused by higher exposure to traumatic events. HIV-infected children with a higher level of social support may be more likely to believe that others would provide the necessary resources to solve the problem when they encounter stress. This may help them redefine the potential harm posed by their stressful situations and prevent or alter maladaptive behavioral responses to stressful events. It is possible that HIV-infected children with low levels of perceived social support may experience heightened perceptions of threats of stressful events resulting in high levels of depressive symptoms. Enhancing perceived social support may, therefore, prevent or decrease depressive symptoms in HIV-infected children.

We also observed a positive correlation between catastrophizing and depression, suggesting that by using the strategy of catastrophizing, HIV-infected children may be more vulnerable to developing and exacerbating depression in response to negative life events than others. Studies have shown that cognitive emotion regulation styles such as self-blaming, catastrophizing, and rumination show strong relationships with internalizing problems [31–33]. Internalizing problems include difficulties that are directed inwards, such as disordered mood, anxiety, and depression [51]. A number of authors have suggested that depressive mood and symptoms could be a consequence of the frequent use of maladaptive emotion regulation [29, 52, 53]. In other words, although dysfunctional attitudes are accessible during stressful life periods, the strategies that individuals use to regulate these cognitions (self-blaming, focus on positives, planning, positive reappraisal, catastrophizing) seem to influence the occurrence of associated depression. In a study of HIV-infected men, Crues et al. [54] observed that an improvement in cognitive coping strategies and reduction in dysfunctional attitudes were closely associated with decreases in depression. Attachment theory states that early traumatic experience, especially with a caregiver, creates an environment which invalidates emotions [55, 56]. In addition, traumatic experiences with a caregiver leads to an insecure attachment style in children. An invalidating environment and insecure attachment can cause individuals to use maladaptive strategies [57, 58]. HIV-infected children often lose their parents or experience inadequate caring relationships with them. According to the theoretical perspective of social learning, emotionally neglected individuals have deficient opportunities to learn adaptive emotion regulation strategies through caregiver modeling [59]. Therefore, they are inclined to evaluate the consequences of negative events using maladaptive strategies. From the perspective of cognitive behavioral theory, the maladaptive appraisal of negative life events may be at the core of depression and anxiety. Maladaptive processing of stressful events can overwhelm patients with negative emotions, thus contributing to clinically significant depression and anxiety [60]. Similar to findings by Noel et al. [27], HIV-infected children facing pressure caused by HIV/AIDS preferring to use the cognitive emotion regulation of catastrophizing to handle stressful situations are susceptible to depression. Based on this study, suggestions for intervention in HIV-infected children may include challenging the maladaptive strategy of catastrophizing.

We also found that social desirability was a major influential factor in depression experienced by Chinese HIV-infected children. Inconsistent, however, with other studies, in our study children having high levels of social desirability reported higher levels of depression. In studies of chronic disease in children, Deirdre et al. suggest that children scoring high on a measure of social desirability reported lower depression and anxiety compared to children scoring low on a social desirability index [15]. One possible explanation, consistent with mainstream education and authority of society, children scoring high on a measure of social desirability respond in a culturally and socially acceptable manner that would obtain approval rather than blame and criticism from their parents, teachers, or peers. Phipps and Steele put forward another possible explanation. They propose that defensiveness and selective reporting may explain the low scores for depression and anxiety when children with a long-term illness are hiding their true feelings and engaging in a pretense of normality [61]. However, in our study, HIV-infected children having high levels of social desirability reported higher levels of depression. We interpret this finding to reflect the possibility that due to lower maturity of cognitive levels, children are typically susceptible to social desirability influences. Studies have suggested that children who are strongly influenced by social desirability may deny negative thoughts and feelings in an effort to normalize their social world [62, 63]. Most importantly, as HIV-infected children, they not only have to face the burden of studying, relationships with peers, and bodily changes, but also pressure coming from the stigma, treatment, and progression of HIV infection. Consequently, for being normal and thus avoid strong controls, overt disapproval, threats of rejection from society and surroundings, they felt it was necessary to suppress negative thoughts and feelings from their own awareness. Due to the potential loss of parents, or poor relationships through caring for their parents, HIV infected children lacking from warmth may respond in a culturally and socially favorable manner rather than one true to themselves in order to be accepted, recognized, and appreciated from teachers and kinsfolk. In the long progress of denying and suppression, HIV-infected children may not correctly release and express their negative emotion. According to the theory of the strategy of emotion regulation, when individuals adopted some form of suppressive emotional regulation to deal with increased subjective negative emotional experience, this eventually leads to depressive disorder [64, 65]. All of the above findings suggest to us that in the daily life of HIV infected children, we can take measures of perceived social support, cognitive emotion regulation and social desirability to address the difficulty of depression. We suggest using this knowledge to decrease the damage caused by depression in HIV-infected children.

## Limitation

Some limitations of this study must be acknowledged. The first limitation is the potential for sampling bias, as the participants in the present study were all recruited from the Henan province where a large number of rural residents were affected with HIV through unhygienic blood collection practices. These children infected by vertical transmission are not representative of all HIV-infected children throughout China. Second, the data are based entirely on self-evaluations, which may have introduced sources of bias and error. The third limitation involves the weak support for causal inference due to the cross-sectional design of this study. Therefore, future studies should include a longitudinal follow-up to determine the relationships between depression and other factors in HIV infected children.

## Conclusions

To conclude, the present study examines factors associated with depression among HIV infected children in China from the psychosocial perspective. Findings suggest that perceived social support has a significant and positive effect on depression in children affected by HIV but that children’s social desirability and one dimension of cognitive emotion regulation (that is, catastrophizing) have a negative impact. Health interventions are warranted to reduce the depression so as to reduce the damage caused by depression in children affected by HIV.

## Abbreviations

HIV: human immunodeficiency virus
AIDS: acquired immune deficiency syndrome
CDI: Children’s Depression Inventory
CERQ: Cognitive Emotion Regulation Questionnaire
MPSS: Multidimensional Scale of Perceived Social Support
SPSS: Statistical Package for Social Sciences
SD: standard deviation
